# Exodontia

**Published:** 1913-10-15

**Authors:** 


EXODONTIA. This is a new book on extracting teeth, written by George B. Winters, D.D.S. Lecturer on Anesthesia in the St. Louis University School of Dentistry, and published by the American Medical Book Co., St. Louis, Mo., It is a large octavo $6\frac{3}{4}$ by 10 inches, containing 430 pages. It is beautifully printed on heavy glazed paper and is very fully illustrated. There are seventeen chapters, as follows: Historical, Instruments, Office Equipment, Anatomical Landmarks, Indications for Extraction, Examination of Mouth, Position of Patient and Operator, Precautionary Measures, Technique for Upper Teeth, Technique for Lower Teeth, Technique for Impacted Teeth, Deciduous and Supernumeraries, Hypercementosis, Accidents, Treatment of Wounds, Hemorrhage, General and Local

Anesthetics. As this statement indicates the subject is presented in a logical manner, and each subject is comprehensively considered. The method of treatment is pedagogically correct and the text for the greater part satisfactory, of course every one will not approve of methods that do not appeal to him as so good as his own practices; as for instance, we use the rotary force largely in breaking the attachment of roots to the aveolar process rather than the luxating movements suggested by the author, and the authors selection of forceps and elevators do not appeal to us as the best. The material of the book however is so excellent and the work is so thoroughly done that we are glad to cordially commend the book, and we want to congratulate the author and publishers also for this most excellent addition to our working material and literary output. It is by all odds the best thing of its kind.

